# Theoretical and Structural Study of Axial Symmetry Ce^3+^ Centers in the BaWO_4_ Single Crystal Doped with Cerium and Codoped with Sodium Ions

**DOI:** 10.3390/ma15165749

**Published:** 2022-08-20

**Authors:** Tomasz Bodziony

**Affiliations:** West Pomeranian University of Technology in Szczecin, Al. Piastów, 17, 70-310 Szczecin, Poland; tbodziony@zut.edu.pl

**Keywords:** electron paramagnetic resonance (EPR), crystal field theory, spin Hamiltonian, tetragonal symmetry, BaWO_4_, Ce^3+^, Na^+^

## Abstract

The spin–Hamiltonian parameters g–factors ($g_{\left|\right|}$ and $g_{⊥}$) of the Ce^3+^ paramagnetic centers in BaWO_4_: Ce and BaWO_4_: Ce, Na single crystals with axial symmetry are investigated using the superposition model (SPM) via complete diagonalization procedure of energy matrix (CDM method). The calculated g–factors are in reasonable agreement with the experimental values. The fitted intrinsic parameters are comparable with data from other publications for rare-earth paramagnetic centers in a similar environment. The angular distortions of the cerium dodecahedron [CeO_8_] are also studied. Structural analysis of paramagnetic centers with axial symmetry through the postulated cerium barium tetrahedron [CeBa_4_] connected via oxygens bridges was carried out. The mechanism of the charge compensation and the role of the second dopant (Na^+^) is also discussed.

## 1. Introduction

The barium tungstate (BaWO_4_, BWO) crystal with a scheelite structure has attracted the interest of several research groups as a potential material for many applications, for example, in solid-state lasers, Raman shifters, stimulated Raman scattering, scintillators, etc. [1,2,3,4]. Generally, the group of tungstate materials like BaWO4, belonging to the ABO4 common group molybdates and tungstates [5], is interesting to scientists because of their optical applications, especially after doping them with alkali metal ions (Me) and/or trivalent rare earth ions (Re^3+^). The rare-earth doped single crystals offer a wide range of optical applications. However, the doping process is not easy and is not always successful. Another ion replaces the host crystal ion with a different valence and ionic radius. The new ions must have an acceptable radius (by the crystal) and require a charge compensation mechanism. Structural defects can provide the charge compensation. Another option is to use a second dopant with proper alkali metal ions. The barium tungstate doped with neodymium (BaWO_4_: Nd were obtained and investigated by I.S. Voronina et al. [6]. The barium tungstate doped with praseodymium (BaWO_4_: Pr^3+^) was analyzed by L. Jinsheng et al. [7]. The barium tungstate doped with praseodymium and codoped with sodium (BaWO_4_: Pr^3+^, Na^+^) were examined by S. Kaczmarek et al. [8]. The CaWO_4_ single crystal doped with Eu^3+^ and codoped with Na^+^ were also investigated [9]. Another interesting paper is an article written by A. K. Kunti et al. focused on BaWO_4_ doped with Dy^3+^ and codoped with K^+^ (Ba_1-x-y_Dy_x_K_y_WO_4_ (x = 0.10; y = 0.05)) [10]. A highly doped BaWO_4_ single crystal is investigated, and research concentrates on the optical and XRD measurements. This paper also discusses the issue of charge compensation in highly doped and codoped barium tungstate (BaWO_4_: Dy^3+^, K^+^) [10]. 

We will focus on another pair of dopant and codopant in a BaWO_4_ crystal: cerium (Ce^3+^) and sodium (Na^+^). S. Kaczmarek et al. published the article about BaWO_4_: Ce single crystal codoped with sodium ions [11]. The four samples of the BaWO_4_ single crystal doped with cerium and codoped with sodium at different dopant concentrations were investigated by the EPR measurements [11]. The EPR measurements (electron paramagnetic resonance) are a very sensitive method for studying the paramagnetic ion and its environment. The paramagnetic ion, like the rare-earth ion (Cr^3+^), acts as a probe inserted into the host network. Knowing the position of the dopant ion and its surroundings is very important for future applications of the doped crystal, for example, optical applications, like laser action. However, a theoretical analysis must be added to the experimental studies, i.e., EPR measurements, to obtain structural data. 

Two types of paramagnetic centers were detected in the studied monocrystals BaWO_4_: Ce and BaWO_4_: Ce, Na: centers with axial symmetry and centers with low symmetry (C_2_) [11]. The paramagnetic Ce^3+^ centers with axial symmetry in the BaWO_4_ single crystal were the subject of theoretical investigations in our two previous papers [12,13]. In the first paper, the theoretical and structural analysis of centers with axial symmetry was performed based on a simplified Newman model [12]. The strongest parenetic center (Ce^3+^) with axial symmetry occurring in all studied samples (BaWO_4_: Ce and BaWO_4_: Ce, Na single crystals) was the subject of the second paper [13], and the full superposition model (SPM) was used. The present paper is a continuation and conclusion of those studies: the cerium (Ce^3+^) paramagnetic centers with axial symmetry in BaWO_4_: Ce and BaWO_4_: Ce, Na single crystals. 

There are many very interesting crystals with wolframite and scheelite structures like BaWO_4_, CaWO_4_, PbWO_4_, SrWO_4_, and so on. They have common structure types for ABO_4_ compounds [5]. The barium tungstate (BaWO_4_) crystal (or its unit cell) is characterized by a tetragonal space group with $C_{4h}^{6}$ ($I4_{1}/a$) symmetry [14]. Lattice parameters are: a = b = 5.6148 Å, c = 12.721 Å [5,14]. The structural characteristics of the BWO unit cell are barium dodecahedrons [BaO_8_] and tungstate tetrahedrons [WO_4_]. Ions Ba^2+^–Ba^2+^ and Ba^2+^–W^6+^ are connected via oxygens bridges (O^2−^). Ba^2+^ and W^6+^ sites have S_4_ point symmetry. The barium dodecahedron [BaO_8_] is a compound of two rotated oxygens tetrahedrons, where the distances between barium and oxygen ions (Ba^2+^–O^2−^) are equal to 2.7857 Å and 2.8310 Å, respectively. Each barium dodecahedron [BaO_8_] is connected through its edges with four neighboring dodecahedrons [BaO_8_]. The [WO_4_] tetrahedron has a nearby regular shape. However, it exhibits a slightly distorted shape along the **c** (S_4_) axis. The W^6+^–O^2−^ bond length is 1.8230 Å [5]. Each oxygen ion (O^2−^) is bonded to two barium ions (Ba^2+^) and one tungstate ion (W^6+^). In a pure BWO crystal, as in any other, the ions’ positions and their bond angles are strictly fixed, ignoring perturbations such as thermal vibrations of the ions. Nevertheless, the doping process, i.e., the substitution of a dopant ion in place of a barium ion and charge compensation, may change the positions of the oxygen ions, varying the bond lengths and bond angles W–O and Ba–O. As a result of this phenomenon, we obtain distortions of the barium dodecahedron [BaO_8_] and tungstate tetrahedron [WO_4_] [10].

**Figure 1:**
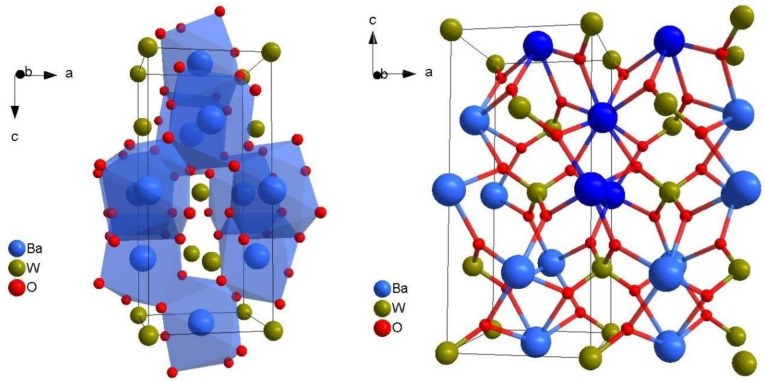
Unit cell structure of BaWO_4_ single crystal: (1) left picture-unit cell with marked barium dodecahedrons [BaO_8_] (2) right picture-double unit cell with marked Ba^2+^-Ba^2+^ and Ba^2+^-W^6+^ connections via oxygens bridges. The five Ba^2+^ ions are highlighted in dark blue color.

Figure 1 shows unit cell structures of BaWO_4_ single crystal viewed approximately along the **b** axis. The left picture presents a standard single unit cell with marked barium dodecahedrons [BaO_8_]. The tungstate tetrahedrons [WO_4_] have not been marked for the clarity of the picture. The right picture shows a double unit cell viewed in the same direction. Ions’ connection Ba^2+^–Ba^2+^ and Ba^2+^–W^6+^ via oxygens bridges are also marked. A double unit cell is not a standard drawing of a unit cell structure. However, important structural elements are sometimes visible only when their unit cell is doubled. Therefore, the important posts of the structure—the five Ba^2+^ ions—are highlighted in dark blue color (Figure 1, right picture). 

Cerium ion (Ce^3+^, 4f^1^ electronic configuration) is the so-called Kramer’s ion. Its ^2^F_5/2_ ground state in tetragonal symmetry is split into tree Kramer’s doublets. Only the lowest doublet is populated, and transitions between its levels are observed in the EPR measurements. Therefore, effective spin is S = 1/2. A literature review has shown that only a few papers exist on Ce^3+^ or other rare-earth (Re^3+^) ions in BWO. There is one interesting paper about erbium ions in barium tungstate in BaWO_4_: Er was found [15] and an article about EPR g-factors for the tetragonal Ce^3+^ centers in YPO_4_ and LuPO_4_ crystals [16]. However, there are many articles about rare earth centers in similar crystal environments. Ytterbium ions (Yb^3+^) in CaWO_4_: Yb single crystal [17], erbium ions Er^3+^ in CaWO_4_: Er and SrWO_4_: Er crystals [18]. Cerium (Ce^3+^) and ytterbium ions and (Yb^3+^) in garnets [19,20]. Erbium (Er^3+^) centers in zircon-type compounds and PbMoO_4_, SrMoO_4_ crystals [21,22]. An interesting article about F-type color centers in BaWO_4_ crystals [23,24]. The current article is based on our previous papers on the cerium centers in BaWO_4_: Ce and BaWO_4_: Ce, Na single crystals [11,12,13]. This study has four BWO crystals: (1) BaWO_4_: 0.5% at. Ce, (2) BaWO_4_: 1.0% at. Ce, (3) BaWO_4_: 0.5% at. Ce, 1.0% at. Na and (4) BaWO_4_: 1.0% at. Ce, 2.0% at. Na [11]. Five paramagnetic centers (Ce^3+^) with axial symmetry and several (more precisely, six) centers (Ce^3+^) with low symmetry (C_2_) were detected in the EPR measurements in the above crystals [11,12]. This work aims to theoretically analyze the spin Hamiltonian parameters of all paramagnetic centers with axial symmetry presented in the BWO monocrystal doped with cerium and codoped with sodium. The second objective is the structural study of these centers in the BWO crystal. We will also deal with the influence of the codopant (Na^+^) on the structure of the cerium centers in BWO crystals. 

**Figure 2:**
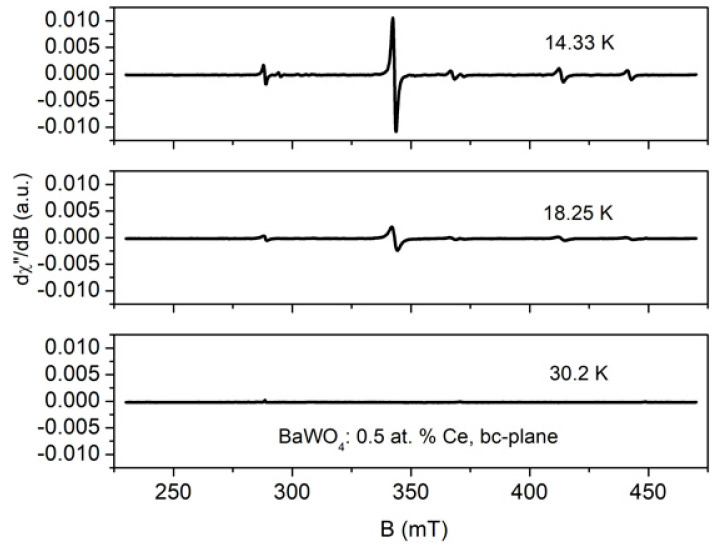
EPR spectra of the BaWO_4_: 0.5% at. Ce recorded in different temperatures [11].

Figure 2 shows examples of the EPR spectra of the BaWO_4_: 0.5% at. Ce single crystal taken at different temperatures. Figure 2 is a reprint of the original figure from the paper S. Kaczmarek et al. published in Crystal in 2019 [11] courtesy of prof. S. Kaczmarek. One can see separate and single resonance lines coming from different paramagnetic centers. That resonance lines disappear at temperatures above 30 K. EPR measurements can be regarded as a very sensitive method of studying the interaction between a paramagnetic ion and its environment. The spin Hamiltonian parameters are obtained directly from the EPR measurements [25]. In order to obtain structural information from spin Hamiltonian parameters, one must use theoretical models. There are two models: (a) superposition model (SPM) [26,27] and/or (b) perturbation methods (PM) up to second order (or higher) [28]. The superposition model (SPM) is used in this paper to obtain structural information about cerium ion (Ce^3+^) and its surroundings in BWO monocrystals from spin Hamiltonian parameters. The obtained results will be analyzed and compared with similar results from other publications. The layout of the article is standard. After the introduction, we have a theoretical part with calculations. Then we have the structural analysis of cerium (Ce^3+^) centers and their surroundings. Finally, we have a discussion and conclusions.

## 2. Theoretical Model and Calculations

It is assumed that the cerium ions (Ce^3+^), like other rare-earth impurities, take the place of the barium ion (Ba^2+^) in the BWO unit cell and preserve its site symmetry (tetragonal, S_4_) [10,11,12,13,15,23,24]. The substitution of Ce^3+^ ion in place of Ba^2+^ requires charge compensation. Barium vacancies compensate for the excess charge ($V_{Ba}^{2-}$). This means that two substitutions ($Ce_{Ba}^{3+}$ or generally rare-earth ions $Re_{Ba}^{3+}$) are balanced by one barium vacancy ($V_{Ba}^{2-}$) [10,11,12,13,15]. The barium vacancy ($V_{Ba}^{2-}$) is not necessarily located in close surroundings of cerium ions. H. Zhang et al. proposed another mechanism for charge compensation through oxygen bridges (O^3−^), leading to F-type color centers in pure (undoped) BaWO_4_ crystal [23,24]. However, it seems that in the case of monocrystalline BWO doped with rare earth ions, this type of compensation is of marginal importance.

Cerium ion (4f^1^ electronic configuration) has a ^2^F_5/2_ ground state and ^2^F_7/2_ excited state in tetragonal symmetry (S_4_). Instead of this type of symmetry, the D_2d_ symmetry approximation is often used because of the small distortion [15,16,18,19,23]. The crystal field with D_2d_ symmetry splits the ground state (^2^F_5/2_) and excited states (^2^F_7/2_) into three and four doublets, respectively. Only the lowest doublet is populated; therefore, the Ce^3+^ ion has an effective spin S = ½, as mentioned earlier. The spin–Hamiltonian for Ce^3+^ ion can be written as [25]:

(1) $$\hat{H}={\hat{H}}_{f}+{\hat{H}}_{SO}+{\hat{H}}_{CF}+{\hat{H}}_{Z}$$

where ${\hat{H}}_{f}$ denotes free ion term, ${\hat{H}}_{SO}=\xi \left(\hat{L}\cdot \hat{S}\right)$ this is the spin-orbit coupling term, with ξ-the spin-orbit coupling parameter, ${\hat{H}}_{CF}$–means a crystal field term, and in ${\hat{H}}_{Z}$ denotes Zeeman interaction. In the case of rare earth ions, we must consider the total angular momentum $\hat{J}$ in the ^2S+1^L_J_ manifold. Therefore the Zeeman term is usually written as [16,19,25]:

(2) $${\hat{H}}_{Z}=g_{J}\;\mu_{B}\;\hat{J}\cdot \vec{H}$$

As a result, we obtain a 14 × 14 energy matrix for cerium ion (Ce^3+^) according to spin Hamiltonian (1). Next, after diagonalization, the eigenvalues (energy levels) of the positions of resonance transitions between energy levels can be calculated in magnetic field units or g factors. In the case of tetragonal, axial symmetry, we have two g factors: g parallel ($g_{\left|\right|}$) to the **Z** axis and g perpendicular ($g_{⊥}$), where axes **Z** is parallel to the crystallographic **c** axes (Figure 1). According to the paper by H.G. Liu et al. [16], the Zeeman term was first added to spin-Hamiltonian. Then the full spin Hamiltonian matrix was diagonalized according to the procedure of the complete diagonalization method (CDM) [16], which was used in the previous paper about the axial center Ce^3+^ in barium tungstate crystal [13]. One can determine only two g factors: g parallel ($g_{\left|\right|}$) and g perpendicular ($g_{⊥}$) for this system. Theoretical calculated values of these g factors are established according to the following formulas:

(3) $$g_{\left|\right|}=\frac{E_{\left|\right|}}{\mu_{B}H_{\left|\right|}}\;g_{⊥}=\frac{E_{⊥}}{\mu_{B}H_{⊥}}$$

It is worth recalling that the parallel direction is determined by the **Z** axis parallel to the crystallographic **c** axis, while any axis perpendicular determines the perpendicular direction to **Z**, say axis **X**. $E_{\left|\right|}$ and $E_{⊥}$ means the Zeeman splitting between the lowest energy doublet obtained by the CDM method matrix from Equation (1) in the magnetic field along the **Z** and the **X** axes, respectively. The crystal field term is a very important term of the spin Hamiltonian for rare earth ions [25]. The crystal field interaction ${\hat{H}}_{CF}$ for Ce^3+^ ion in tetragonal symmetry (D_2d_) can be written in terms of Stevens operator equivalent [25]:

(4) $${\hat{H}}_{CF}=B_{2}^{0}\;C_{2}^{0}+B_{4}^{0}\;C_{4}^{0}+B_{4}^{4}C_{4}^{4}+B_{6}^{0}\;C_{6}^{0}+B_{6}^{4}C_{6}^{4}$$

where parameters $B_{k}^{q},\;k=2,\;4,\;6,\;\left|q\right|\;\leq k$ are the crystal field parameters. In the superposition model (SPM), the crystal field parameters are calculated according to the formula [25]:

(5) $$B_{k}^{q}=\overset{n}{\underset{i=1}{\sum }}{\bar{A}}_{k}\left(R_{0}\right){\left(\frac{R_{0}}{R_{i}}\right)}^{t_{k}}K_{k}^{q}\left(\theta_{i},\;\varphi_{i}\right)$$

The summation proceeds after the nearest ligands surrounding the paramagnetic ion. In our case, it is after eight oxygen ions form a barium dodecahedron [BaO_8_]. Parameters: ${\bar{A}}_{k}\left(R_{0}\right)$ and $t_{k}$ are the intrinsic parameters and the power law exponents, respectively. The parameter $R_{0}$ is a standard metal–ligand distance. The parameters $K_{k}^{q}\left(\theta_{i},\;\varphi_{i}\right)$ are so-called geometric coordination factors, key coefficients to relate the system’s geometry to the crystal field parameters. The formulae for the coefficients are given in papers written by D. J. Newman [26,27]. 

In Equation (5), there are parameters whose values have to be assumed in advance (a priori) and those which can be obtained from crystallographic data of the BaWO_4_ crystals. The dodecahedron [BaO_8_] is formed by two rotated tetrahedrons [BaO_8_]. The structural parameters are azimuthal angles $\varphi_{i}$, the polar angles $\theta_{i}$, the distance $R_{i}^{H}$ between two barium tetrahedrons, i=1, 2. The following values of the structural parameters were taken: $R_{1}^{H}\approx 0.2778\;$[nm], $\theta_{1}\approx 69.05^{0}$, $\;\varphi_{1}\approx -35.16^{0}$, and $R_{2}^{H}\approx 0.2738\;$[nm], $\theta_{2}\approx 143^{0}$, $\;\varphi_{2}\approx -24.41^{0}$, for the first and the second tetrahedron, respectively [29]. For the ionic radii and crystal radii, all positive ions in the doped and codoped barium tungstate (BaWO_4_: Ce and BaWO_4_: Ce, Na) are gathered in Table 1 according to R. D. Shannon [30]. 

The local lattice relaxation arising from the cerium substitution ($Ce_{Ba}^{3+}$) can be satisfactorily estimated from the equation [16,18,19,20]:

(6) $$R_{j}\approx R_{j}^{H}+\frac{r_{I}-r_{H}}{2}$$

where $r_{I}$ and $r_{H}$ are the ionic radii of the cerium ion (impurity) and the barium (host) ion, respectively. We can calculate the last structural parameters. The distances Ce^3+^–O^−^ are equal $R_{1}=0.2608\;\left[\mathrm{nm}\right],\;R_{2}=0.2568\;\left[\mathrm{nm}\right]$, for two tetrahedrons, respectively. All structural data necessary for the calculations are summarized in Table 2.

Finally, someone has to determine the values of other parameters from the SPM model (Equation (5)). We used typical values for rare earth ions in a similar oxygen environment. The power law exponents were determined as $t_{2}\approx 5$, $t_{4}\approx 6$, $t_{6}\approx 10$ [16,26,27,31,32]. Last but not least, the spin-orbit coupling parameter is assumed ξ ≈606 [cm^−1^]. This is the mean value of the spin-orbit coupling parameter obtained for the same cerium dodecahedrons [CeO_8_] in YPO_4_, and LuPO_4_ crystals of a similar structure by H.G. Liu et al. [16]. It is worth mentioning that similar values of the spin–orbit coupling were obtained for Ce^3+^ ions in Cs_2_NaYCl_6_ crystal $\xi_{4f}\approx 624.1$ [cm^−1^] [33] and in various fluoride compounds $\xi_{4f}\approx 614.9$ [cm^−1^] [34]. 

The calculations were done for five paramagnetic cerium centers (Ce^3+^) with axial symmetry in the four doped barium tungstate crystals. The values of g–factors: g perpendicular ($g_{⊥}$) and g parallel ($g_{\left|\right|}$) for all five axial centers are collected in Table 3 [13]. 

One paramagnetic centrum with axial symmetry is recorded in all four samples, with one center in BaWO_4_: 1.0% at. Ce, two centers in BaWO_4_: 0.5% at. Ce, 1.0% at. Na and one center detected in BaWO_4_: 1.0% at. Ce, 2.0% at. Na. The calculation procedure (based on the CDM method) consisted of checking which intrinsic parameters ${\bar{A}}_{2}\left(R_{0}\right),\;{\bar{A}}_{4}\left(R_{0}\right),\;{\bar{A}}_{6}\left(R_{0}\right)$ could obtain the best approximation to the experimental g factors Equations (4) and (5). As a result, we obtain the values of the intrinsic parameters gathered in Table 4. In the next stage, one checked how changes in the position of oxygen ligands, i.e., change of the polar angles $\theta_{i}$, improve the agreement between theoretical and experimental g factors. It turned out that small changes in the polar angles $\theta_{i}$ are enough ($\Delta \theta_{1}=$ $\Delta \theta_{2}\leq 1.0^{0}$).

Center no. 1 is the strongest paramagnetic (Ce^3+^) center with axial symmetry presented in all BaWO_4_ samples. Intrinsic parameters with values characterize this center: ${\bar{A}}_{2}\left(R_{0}\right)\approx 861\;\left[{\mathrm{cm}}^{-1}\right]$, ${\bar{A}}_{4}\left(R_{0}\right)\approx 21\;\left[{\mathrm{cm}}^{-1}\right]$, ${\bar{A}}_{6}\left(R_{0}\right)\approx 1\;\left[{\mathrm{cm}}^{-1}\right]$. Other values of the intrinsic parameters for center no 1 are given in the earlier work [13]. The reason for the discrepancy is that we previously put some lower bounds on the values of the intrinsic parameters; for example, we assumed that ${\bar{A}}_{6}\left(R_{0}\right)\geq 2.0\;\left[{\mathrm{cm}}^{-1}\right]$. These limitations seemed to be right based on the literature data [15,16,17,18,19,20,21,22,23,24]. In the current fitting procedure, we have extended the search range and left only the simplest conditions: ${\bar{A}}_{2}\left(R_{0}\right)>{\bar{A}}_{4}\left(R_{0}\right)>{\bar{A}}_{6}\left(R_{0}\right)$ and non-zero values of the sixth–rank parameter: ${\bar{A}}_{6}\left(R_{0}\right)>0$. It seems, that this approach is correct. One can see from Table 4 that the intrinsic parameters six order and fourth order are almost the same for all centers: ${\bar{A}}_{6}\left(R_{0}\right)\approx 1\;\left[{\mathrm{cm}}^{-1}\right]$, ${\bar{A}}_{4}\left(R_{0}\right)\approx 21÷24\;\left[{\mathrm{cm}}^{-1}\right]$. Only the intrinsic parameters the second order are significantly changed in the range ${\bar{A}}_{2}\left(R_{0}\right)\approx 861\;÷983\;\left[{\mathrm{cm}}^{-1}\right]$, for center no 1 and center no 5, respectively (Table 4). It has been shown that small angular distortion of the polar angles ($\Delta \theta =\Delta \theta_{1}=$ $\Delta \theta_{2}$) results in a significant improvement in the fit of the theoretical calculated parameters of the spin Hamiltonian to the experimental values. However, it turned out that slight changes in the polar angles are sufficient ($\Delta \theta \leq 1.0^{0}$). It would indicate that the structure of the cerium dodecahedron [CeO_8_] is rather “rigid”, and not very susceptible to shifts of the dopant ion.

First, let us consider how these five paramagnetic centers (Ce^3+^) of axial symmetry arise in the BaWO_4_ monocrystals doped with cerium or doped with cerium and codoped with sodium. Why are there five of them? How does the charge compensation mechanism work? How does it affect the paramagnetic center and its surroundings?

## 3. Structural Analysis of the Cerium (Ce^3+^) Centers in the BWO Single Crystals

**Figure 3:**
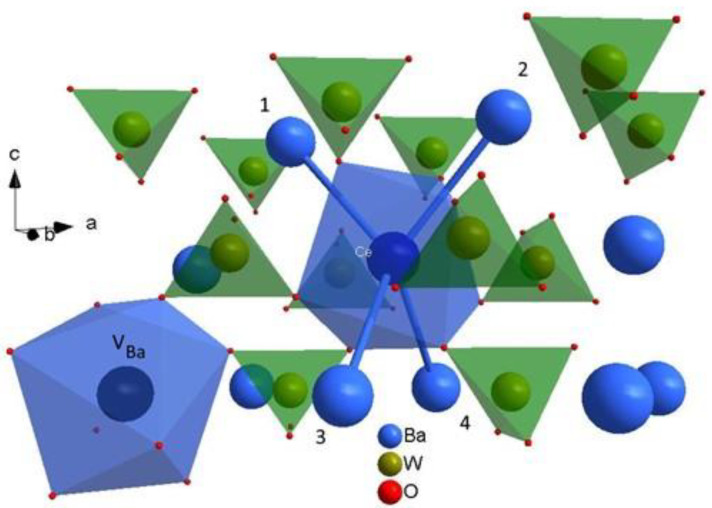
Part of the unit cell structure of a BaWO_4_ single crystal with marked [CeBa_4_] tetrahedron (1 – 4 Ba^2+^ ions and Ce^3+^ ion in the middle). The cerium dodecahedron [CeO_8_], the barium vacancy dodecahedron [V_Ba_O_8_], and tungstate tetrahedrons [WO_4_] are also shown.

**Figure 4:**
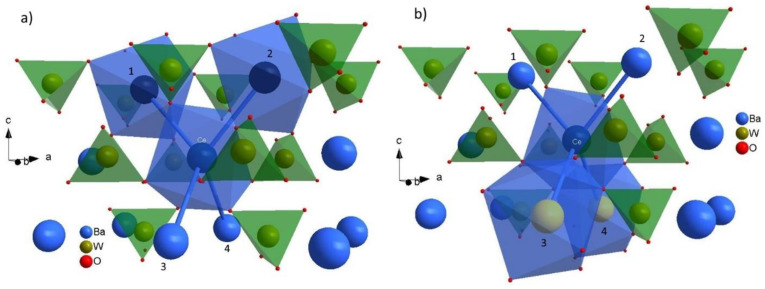
[CeBa_4_] tetrahedron in two cases. (**a**) The two barium vacancies ($V_{Ba}^{2-}$) in the barium sites 1 and 2 are marked with a dark blue color (the left picture). (**b**) The two sodium ions in the barium sites 3 and 4 ($Na_{Ba}^{+}$) are marked with yellow color (the right picture).

Cerium ions (Ce^3+^), or other rare-earth ions, are assumed to take the place of barium ions (Ba^2+^) in the BaWO_4_ single crystal. Barium vacancies achieve compensation for the excess charge ($V_{Ba}^{2-}$). One barium vacancy ($V_{Ba}^{2-}$) balances two substitutions of a cerium ion for a barium ion ($Ce_{Ba}^{3+}$) [10,11,12,13,15]. Let us take a look at the unit cell of BaWO_4_ single crystal. The interesting structure of the unit cell, the five Ba^2+^ ions, is highlighted in dark blue color (Figure 1, right picture). Four barium ions surround each barium ion. These five barium ions (Ba^2+^) form an important part of the crystal structure, a tetrahedron of barium ions. If the cerium ion is substituted in place of the barium ion, the tetrahedron [CeBa_4_] is obtained (Figure 3). There is an important difference between the tungstate tetrahedron [WO_4_] or the barium dodecahedrons [BaO_8_] and tetrahedron [CeBa_4_]. In the first two structures, there is a direct interaction between positive (Me^n+^) and negative ions (O^2−^). In the [CeBa_4_] tetrahedron, there is only an indirect interaction. The positive ions interact with each other through the negative oxygen ions (O^2−^), or oxygen bridges. This tetrahedron [CeBa_4_] is shown in Figure 3 together with the tungstate tetrahedrons [WO_4_]. The cerium dodecahedron [CeO_8_] and barium vacancy dodecahedron [V_Ba_O_8_] are also shown in Figure 3. The barium vacancy ($V_{Ba}^{2-}$) is marked by a black circle. One can see from Figure 3 that the barium vacancy ($V_{Ba}^{2-}$) and the cerium ion (Ce^3+^) do not interact with each other, either directly or indirectly. Therefore, the cerium center maintains axial symmetry. What happens when the “top” two or two “bottom” barium sites in the tetrahedron [CeBa_4_] are occupied by barium vacancies or codopant ions? This structure is presented in Figure 4. The left figure shows a situation where the “top” two barium sites (1 and 2, dark blue color) are occupied by barium vacancies ($V_{Ba}^{2-}$) (Figure 4a). The right picture shows a situation where the “bottom” two barium sites (3 and 4, yellow color) are occupied by codopant ions; in this case, sodium ions ($Na_{Ba}^{+}$) (Figure 4b).

We have the following possibilities: a pair of barium vacancies ($V_{Ba}^{2-}$) in the “upper” (1, 2) and “lower” (3, 4) position and a pair of sodium ions ($Na_{Ba}^{+}$) in the “upper” and “lower” position (Figure 3). It is easy to see that the symmetry of the cerium ion and its surroundings is preserved in all cases. This gives four cerium paramagnetic centers with axial symmetry. Plus, one center discussed earlier (see Figure 3). In total, we have five paramagnetic centers with axial symmetry that have been detected experimentally [11,12]. There is another possibility of a center with axial symmetry. Occupancy of all four sites in barium tetrahedron by barium vacancies (*V_Ba_*) or sodium ions ($Na_{Ba}^{+}$). However, this possibility is purely theoretical for low doped crystals. The probability of such an arrangement is extremely low, and therefore, such a center would be undetectable. 

If mixed pairs occupy the top or bottom site pairs in barium tetrahedron (barium vacancies ($V_{Ba}^{2-}$) and sodium ions ($Na_{Ba}^{+}$) pair) this reduces the symmetry of the system to C_2_ symmetry [10,11,13,15,16]. One substitution in the barium tetrahedron, whether by barium vacancies (*V_Ba_*) or sodium ions ($Na_{Ba}^{+}$) also leads to a decrease in symmetry. It seems appropriate to limit the number of substitutions in the tetrahedron [CeBa_4_] to a maximum of two, or maximally three, for the same reasons. This limits the number of low symmetry centers (C_2_) detected, for example, in EPR measurements. The number of resonance lines recorded in EPR measurements is obviously lower than theoretical estimates. There are insufficient dopant ions to fill all possibilities in low doping cases. Resonance lines (or transitions) can be very weak or overlap.

The second dopant, sodium ions (Na^+^), plays a very important role in forming paramagnetic cerium centers in the BaWO_4_ single crystal. Several pairs of rare-earth and alkali metal ions exist in BaWO_4_ single crystals or others with similar structures. The CaWO_4_ red phosphors with codoping of Eu^3+^ and Na^+^ [9]. S. M. Kaczmarek et al. describe BaWO_4_: Pr^3+^ single crystals codoped with Na^+^ [8]. A. K. Kunit et al. focus on another pair of dopants: dysprosium (Dy^3+^) and potassium (K^+^) in highly doped BaWO_4_ [10]. The cerium (Ce^3+^) and sodium (Na^+^) in BaWO_4_ in a low doping concentration is the subject of several articles [11,12,13]. The crystal radii of the cerium (Ce^3+^) and sodium (Na^+^) ions are respectively: 0.1283, and 0.132 [nm]. The crystal radii of barium ion (Ba^3+^) is equal 0.156 [nm] (Table 1) [30]. The sodium ion is slightly larger than the barium ion (~3 %). The ions of both dopants are much smaller than the host ion (Ba^3+^), about ~18 % and ~15 % for (Ce^3+^) and sodium (Na^+^) ions, respectively. It seems that the second dopant works in several ways. Sodium ions (Na^+^) make it easier for barium ions (or other rare earth dopants) to enter the host crystal because they facilitate the charge compensation mechanism. The simultaneous substitution of sodium and cerium ions in place of barium ions ($Na_{Ba}^{+}\;and\;Ce_{Ba}^{3+}$ versus $2\cdot Ba^{2+}$) eliminates the charge compensation problem. Therefore, sodium ions help barium ions enter the crystal lattice and increase the number of barium ions in the lattice. In addition, the substitution of sodium ions ($Na_{Ba}^{+}$) through charge compensation can affect the surroundings of the barium ion and its symmetries. Sodium ions significantly increase the number of paramagnetic centers and resonance lines (and the optical efficiency), as shown in the case of BaWO_4_ crystal. It seems that adding a second and appropriately selected dopant is a useful and inexpensive process to influence the number of rare earth ions in the host lattice and their symmetries. This is of great importance for future applications of these crystals doped with rare-earth elements such as cerium (Ce^3+^), erbium (Er^3+^), ytterbium (Yb^3+^), praseodymium (Pr^3+^), for example, in optical devices [1,2,3,4,6,7].

## 4. Discussion and Conclusions

This paper is focused on the barium tungstate monocrystals doped with cerium and codoped with sodium. There are four crystals: (a) BaWO_4_: 0.5% at. Ce, (b) BaWO_4_: 1.0% at. Ce, (c) BaWO_4_: 0.5% at. Ce, 1.0% at. Na and (d) BaWO_4_: 1.0% at. Ce, 2.0% at. Na [11,12,13]. Our analysis focuses on five paramagnetic cerium (Ce^3+^) centers of axial symmetry detected in these crystals by EPR measurements. The spin Hamiltonian parameters of these centers, the values of g – factors, g perpendicular ($g_{⊥}$) and g parallel ($g_{\left|\right|}$), are collected in Table 3. The Newman superposition model (SPM model) and complete diagonalization method (CDM) were used to analyze the parameters of the spin Hamiltonian (g factors) [16,17,18,19,20,26,27]. The first and the strongest cerium (Ce^3+^) centrum with axial symmetry in the BaWO_4_ single crystal was analyzed with the same method earlier [13]. The results of the fit procedures there are the intrinsic parameters ${\bar{A}}_{2}\left(R_{0}\right)$,$\;{\bar{A}}_{4}\left(R_{0}\right)$, ${\bar{A}}_{6}\left(R_{0}\right)$ for all five centres are summarized in Table 4. 

We find only one paper for cerium centers (Ce^3+^) in a similar environment [16]. The intrinsic parameters ${\bar{A}}_{2}\left(R_{0}\right)\approx 290,\;{\bar{A}}_{4}\left(R_{0}\right)\approx 48,\;{\bar{A}}_{6}\left(R_{0}\right)\approx 45\;\left[{\mathrm{cm}}^{-1}\right]$ are calculated for LuPO_4_: Ce and YPO_4_: Ce single crystals with zircon structure by H.G. Liu et al. [16]. For erbium (Er^3+^) centers in BaWO_4_ and CaWO_4_, SrWO_4_ crystals, Wu Shao-Yi et al. obtained the following values of intrinsic parameters ${\bar{A}}_{2}\left(R_{0}\right)\approx 400,\;{\bar{A}}_{4}\left(R_{0}\right)\approx 50,\;{\bar{A}}_{6}\left(R_{0}\right)\approx 17\;\left[{\mathrm{cm}}^{-1}\right]$ [21,22]. There are many papers on rare-earth impurities, like Er^3+^, Yb^3+^, and Nd^3+^ in oxide and fluoride crystals with similar symmetry. In general, we can say that the intrinsic parameters of the second, fourth, and sixth order satisfy the inequality ${\bar{A}}_{2}\left(R_{0}\right)\;\gg {\bar{A}}_{4}\left(R_{0}\right)\gg \;{\bar{A}}_{6}\left(R_{0}\right)$. The values of the second rank intrinsic parameter (${\bar{A}}_{2}\left(R_{0}\right)$) are of the order of several hundred, even up to 1200 [cm^−1^] [35]. The lower limit of the sixth rank intrinsic parameter is a few inverse centimeters (${\bar{A}}_{6}\left(R_{0}\right)\approx 2\;\left[{\mathrm{cm}}^{-1}\right])$) [35]. Therefore we can say, that calculated values of the intrinsic parameters for cerium centers with axial symmetry in the BaWO_4_ single crystal are acceptable, and they are within limits reported in the literature. The same is true for the angular distortions of the polar angles. The value of the angular distortion is small is not greater than one degree ($\Delta \theta \;\leq 1^{0}$). 

Cerium (Ce^3+^) and sodium ions (Na^+^) substitute in place of the barium ion (Ba^2+^) in the BaWO_4_ single crystals [10,11,12,13,15,23,24]. The substitution of barium ions in place of tungsten ions (W^6+^) is not excluded under certain circumstances but is very rare if it happens [12]. Its surroundings are key to forming cerium paramagnetic centers with axial and other symmetries. The important structural part of the unit cell is the four closest barium ions, which, together with the cerium ion, form the barium tetrahedron [CeBa_4_] (Figure 3 and Figure 4). The cerium ion (Ce^3+^) and barium ions (Ba^2+^) interact with each other indirectly through oxygen ions (O^2-^). Barium tetrahedron [CeBa_4_] and the charge compensation mechanism via barium vacancies ($V_{Ba}^{2-}$) explain why five cents with axial symmetry is observed. The first and the strongest cerium center (No. 1, Table 4), found in all samples tested, occurs when the barium vacancy ($V_{Ba}^{2-}$) appears outside the tetrahedron without affecting the cerium ion in any way (Figure 3). Another two cerium centers of axial symmetry arise when the bar vacancies ($V_{Ba}^{2-}$) occupy two positions at the “top”, or “bottom” of the tetrahedron [CeBa_4_] (Figure 4a). The last two paramagnetic centers with axial symmetry are associated with codopant-sodium ions (Na^+^)—occupying barium ion sites at the “top” or “bottom” of the tetrahedron (Figure 4b). An odd number of substitutions or mixed and/or not symmetrical substitutions (barium vacancies ($V_{Ba}^{2-}$) and sodium ions ($Na_{Ba}^{+}$)) in the barium tetrahedron [CeBa_4_], leads to a reduction in the symmetry of the system and the formation of a paramagnetic center with the lowest symmetry (C_2_). A very important role is played by the second dopant, e.g., sodium ions (Na^+^). Generally, one can say that the sodium ion ($Na_{Ba}^{+}$) facilitates the entry of the cerium ion ($Ce_{Ba}^{3+}$) into the crystal lattice. In addition, the same charge compensation mechanism makes that the ions: cerium ion ($Ce_{Ba}^{3+}$), sodium ($Na_{Ba}^{+}$) and vacancies ($V_{Ba}^{2-}$) “like” to stay together. If it were otherwise, we would observe only one center with axial symmetry (Figure 3). We recorded yet five centers with axial symmetry in EPR measurements. Therefore, sodium ions also affect the surrounding cerium ion.

This is a very important and interesting subject. New articles are constantly being written on this and similar subjects. It is worth mentioning a few new papers like [36,37]. Although cerium centers with axial symmetry in BaWO_4_: Ce and BaWO_4_: Ce, Na seem to be a very specialized issue, they touch on a very important problem for practical applications of barium tungstate crystals or, more generally, ABO_4_ scheelite compounds doped with rare earth ions: Er, Yb, Pr, Gd, Nd,… Pairs of cerium (Ce^3+^) and sodium (Na^+^) are studied in this work. A. K. Kunti et al. studying the other pair: Dy^3+^ and K^+^ in highly doped BaWO_4_, found that charge compensation improved the color point and increased the quantum efficiency by about 2/3 [10]. Nowadays, rare earth compounds are expensive and important materials of strategic importance. A suitable, cheaper, and more available dopant at least facilitates the entry of rare earth ions into the host crystal, increasing optical efficiency. This can give optically better crystals, lower cost, or both. The process of doping with rare earth elements and codoped with, for example, alkali metal ions and various important monocrystals are worth studying for both theoretical and practical reasons.

## Figures and Tables

**Table 1:** Ionic and crystal radii.

[nm]	Ba^2+^ 8 Coordinate	W^6+^ 6 Coordinate	Ce^3+^ 8 Coordinate	Na^+^ 8 Coordinate
Ionic radii [nm]	0.142	0.060	0.1143	0.118
Crystal radii [nm]	0.156	0.074	0.1283	0.132

R. D. Shannon [30].

**Table 2:** The structural data for cerium dodecahedron [CeO_8_] making of two tetrahedrons in the BaWO_4_ single crystal [13,29].

	$R_{i}$ [nm]	$R_{i}^{H}$ [nm]	$\theta_{i}$ [0]	$\varphi_{i}$ [0]
i = 1	0.2608	0.2778	69.05	−35.16
i = 2	0.2568	0.2738	143.00	−24.41

**Table 3:** The values of g-parameters of Ce^3+^ centers with axial symmetry in the BaWO_4_: Ce and BaWO_4_: Ce, Na single crystals [13].

No. Center	Samples	$g_{\left|\right|}$	$g_{⊥}$
1	All four samples	1.506 (1)	2.712 (2)
2	1.0% at. Ce	1.365 (20)	2.390 (10)
3	0.5% at. Ce, 1.0% at. Na	1.450 (20)	2.650 (10)
4		1.485 (20)	2.680 (10)
5	1.0% at. Ce, 2.0% at. Na	1.460 (20)	2.690 (10)

**Table 4:** The best fitting results of the intrinsic parameters ${\bar{A}}_{2}\left(R_{0}\right),\;{\bar{A}}_{4}\left(R_{0}\right),\;{\bar{A}}_{6}\left(R_{0}\right)$ and the angular distortion $\Delta \theta \;{[}^{0}]$ for paramagnetic centers in the BaWO_4_: Ce and BaWO_4_: Ce, Na single crystals with axial symmetry.

No. Center	${\bar{A}}_{2}\left(R_{0}\right)$ [cm^−1^]	${\bar{A}}_{4}\left(R_{0}\right)$ [cm^−1^]	${\bar{A}}_{6}\left(R_{0}\right)$ [cm^−1^]
1	860.7	20.8	0.9
2	863.5	21.2	0.9
3	956.0	24.0	1.0
4	926.0	23.0	1.0
5	983.0	24.0	1.0

## Data Availability

The study did not report any data.
